# Predictors and Trends of New Permanent Pacemaker Implantation: A Subanalysis of the International Navitor IDE Study

**DOI:** 10.1016/j.shj.2024.100293

**Published:** 2024-03-23

**Authors:** Ibrahim Sultan, Michael J. Reardon, Lars Søndergaard, Bassem Chehab, Dave Smith, Antony S. Walton, Stephen G. Worthley, Ganesh Manoharan, Gerald Yong, Hasan Jilaihawi, Federico Asch, Nicholas Bates, Gregory P. Fontana

**Affiliations:** a Division of Cardiac Surgery, Department of Cardiothoracic Surgery, Heart and Vascular Institute, University of Pittsburgh Medical Center, Pittsburgh, Pennsylvania, USA; b Department of Cardiovascular Surgery, Houston Methodist DeBakey Heart and Vascular Center, Houston, Texas, USA; c Structural Heart Medical Affairs, Abbott Medical, Santa Clara, California, USA; d Department of Cardiology, Ascension Via Christi Hospital, University of Kansas, Wichita, Kansas, USA; e Morriston Hospital, Swansea Bay University Health Board, Swansea, UK; f Department of Interventional Cardiology, Alfred Hospital, Melbourne, Victoria, Australia; g Monash University, Melbourne, Victoria, Australia; h Department of Cardiology, Macquarie University Hospital, Macquarie Park, New South Wales, Australia; i Regional Cardiology Centre, Royal Victoria Hospital, Belfast, UK; j Department of Cardiology, Fiona Stanley Hospital, Murdoch, Western Australia, Australia; k Department of Cardiology, Cedars-Sinai Medical Center, Los Angeles, California, USA; l Cardiovascular Core Labs, MedStar Health Research Institute and Georgetown University, Washington, District of Columbia, USA; m Structural Heart Clinical Affairs, Abbott Medical, St. Paul, Minnesota, USA; n Cardiovascular Institute, Hospital Corporation of America, Los Robles Regional Medical Center, Thousand Oaks, California, USA

**Keywords:** Aortic stenosis, Conduction abnormality, Navitor, Pacemaker, Transcatheter aortic valve implantation, Transcatheter aortic valve replacement

## Abstract

**Background:**

The Navitor Investigational Device Exemption (IDE) study is a prospective, multicenter, global study assessing the safety and effectiveness of the Navitor valve in a population with severe, symptomatic aortic stenosis who are at high and extreme surgical risk. The impact of pre-existing conduction abnormalities and implantation technique on new permanent pacemaker implantation (PPI) for the Navitor platform is not fully understood. Therefore, the goal of this analysis was to investigate the associations between patient and procedural factors and the 30-day new PPI rate.

**Methods:**

A total of 260 patients who underwent implantation of a Navitor valve in the Navitor IDE study were reviewed. Patients with preprocedural permanent pacemakers (n = 28) were excluded. Baseline risk factors were assessed for statistical significance. Multivariable logistic regression analyses were performed to identify independent predictors of new PPI.

**Results:**

Mean age of the pacemaker-naïve population was 83.3 ± 5.2 years, 58.6% were female, average Society of Thoracic Surgeons score was 3.8% ± 1.9%, median frailty score was 1 (interquartile range 1, 2), and 17.7% were deemed at extreme surgical risk. Pre-existing first-degree atrioventricular block and right bundle branch block significantly increased the risk of new PPI postimplantation, whereas left bundle branch block did not. Membranous septum length in relation to noncoronary cusp implant depth was a significant predictor of new PPI, with higher rates of new PPI observed when noncoronary cusp implant depth exceeded membranous septum length. Analysis of implant depth alone revealed deeper implants were associated with a higher rate of new PPI, regardless of patient baseline conduction abnormality.

**Conclusions:**

The 30-day rate of new PPI in the Navitor IDE study is associated with patient pre-existing baseline conduction disturbances and implantation depth.

## Introduction

For elderly patients with symptomatic severe aortic stenosis, transcatheter aortic valve replacement (TAVR) is a safe and effective alternative to surgical aortic valve replacement in all surgical risk groups. However, a common post-TAVR complication is the need for new permanent pacemaker implantation (PPI). PPI post-TAVR has been associated with poor outcomes, such as a higher rate of mortality and increased hospitalizations due to heart failure.^1^^,^^2^ Factors that contribute to new PPI include intrinsic patient characteristics such as pre-existing conduction abnormalities and cardiac anatomical characteristics like length of the membranous septum (MS), valve type (self-expanding vs. balloon expandable), and procedural factors such as implant depth.^3^^,^^4^

The Navitor valve (Abbott Structural Heart, Minneapolis, MN, USA) is a next-generation, self-expanding TAVR device. In the Navitor IDE study (also known as PORTICO NG study, Evaluation of the Portico NG [Next Generation] Transcatheter Aortic Valve in High and Extreme Risk Patients With Symptomatic Severe Aortic Stenosis), which supported Food and Drug Administration approval, the rate of new PPI was 19.0%.^5^ As operators become more experienced with commercially available TAVR systems and as implant techniques are refined, the expectation is that the 30-day rate of new PPI post-TAVR may be expected to decrease, as has been observed with other self-expanding valve platforms.^6^^,^^7^ This report seeks to provide a detailed analysis of patient, anatomical, and procedural factors contributing to the rate of new PPI with the Navitor valve in the Navitor IDE study.

## Methods

### Study Background

The Navitor IDE study (ClinicalTrials.gov: NCT04011722) was initiated to assess the safety and effectiveness of the Navitor TAVR System, which includes the use of the FlexNav delivery system (Abbott Structural Heart, Minneapolis, MN, USA) for valve delivery, in a population at high and extreme surgical risk. The design of the study, procedural aspects, and safety and hemodynamic outcomes through 1 year have been described in detail previously.^5^^,^^8^

### Statistical Methods

Baseline characteristics and medical history were summarized using descriptive statistics. Fisher’s exact method was used to compare patients with or without pre-existing conduction abnormalities that received a pacemaker within 30 days after TAVR and was also used to compare the association of implant depth with the need for new PPI. Implant depth analysis subgroups were defined relative to the target implant depth of 3 mm (2-4 mm), recommended per the Instructions for Use to minimize PPI.

Implant depths <2 mm were considered ‘shallow’, depth 4 to 7 mm were ‘deep’, and depth >7 mm was ‘very deep’, similar to analyses performed by Jilaihawi et al. 2019.^3^ Implant depth was site-reported based on distance from the base of the noncoronary cusp (NCC) to the inflow edge of the deployed Navitor stent frame on postimplant angiography. Subannular MS length was measured by a dedicated computed tomography core laboratory.^3^

Multivariable logistic regression analyses were performed using a stepwise selection process (entry: *p* <0.15, stay: *p* < 0.05) to identify independent predictors of new PPI. Analyses were performed using SAS software version 9.4 (SAS Institute, Cary, North Carolina).

## Results

### Patient Disposition

A total of 260 patients underwent implantation with a Navitor valve between September 2019 and August 2022. Twenty-eight patients had a pre-existing permanent pacemaker and were excluded from the analysis. Therefore, 232 naïve pacemaker patients were assessed for new PPI in this report.

### Baseline Characteristics

Baseline clinical and electrocardiographic variables of the pacemaker-naïve population are summarized in Table 1. The mean age was 83.3 ± 5.2 years, and 58.6% of patients were female. The mean Society of Thoracic Surgeons Predicted Risk of Operative Mortality score was 3.8% ± 1.9% and the median frailty was 1 (interquartile range 1, 2). Sixty-four of the 232 (27.6%) PPI naïve patients at baseline had at least one of the following conduction abnormalities: first-degree atrioventricular (AV) block, second-degree AV block (Type I or II), right bundle branch block (RBBB), left bundle branch block (LBBB), left anterior fascicular block (LAFB); 13 patients (5.6%) had 2 or more abnormalities. Common cardiac arrhythmias and conduction abnormalities included atrial fibrillation (23.3%), RBBB (12.1%), first-degree AV block (9.9%), and LBBB (6.5%). Baseline conduction abnormalities (listed above) were more common in patients who received a new PPI, except for LBBB.

**Table 1:** Baseline characteristics

Baseline variable	Total (N = 232)	New PPI (N = 44)	No PPI (N = 188)
Baseline clinical variables			
Age, y	83.3 ± 5.2	83.8 ± 6.1	83.1 ± 5.0
Sex, female	58.6%	59.1%	58.5%
STS-PROM Score, %	3.8 ± 1.9	3.9 ± 2.2	3.8 ± 1.9
Total Frailty Score			
Median (Q1, Q3)	1 (1, 2)	1 (1, 2)	1 (1, 2)
Baseline electrocardiographic variables			
Persistent atrial fibrillation	23.3%	29.5%	21.8%
AV conduction abnormality∗	33.7%	60.5%	25.0%
Presence of first-degree AV block	9.9%	25.0%	6.4%
Presence of second-degree AV block - Type I	0.9%	2.3%	0.5%
Presence of second-degree AV block - Type II	0.4%	2.3%	0%
Presence of third-degree AV block	0%	0%	0%
Presence of LAFB	3.9%	9.1%	2.7%
Presence of LBBB	6.5%	2.3%	7.4%
Presence of RBBB	12.1%	29.5%	8.0%

Abbreviations: AV, atrioventricular; LAFB, left anterior fascicular block; LBBB, left bundle branch block; PPI, permanent pacemaker implantation; RBBB, right bundle branch block; STS-PROM, Society of Thoracic Surgeons Predicted Risk of Operative Mortality.

∗ Conduction abnormalities are not mutually exclusive Values are mean ± SD or n (%) that reflect missing values.

### Conduction Abnormality Correlation to New PPI

**Figure 1:**
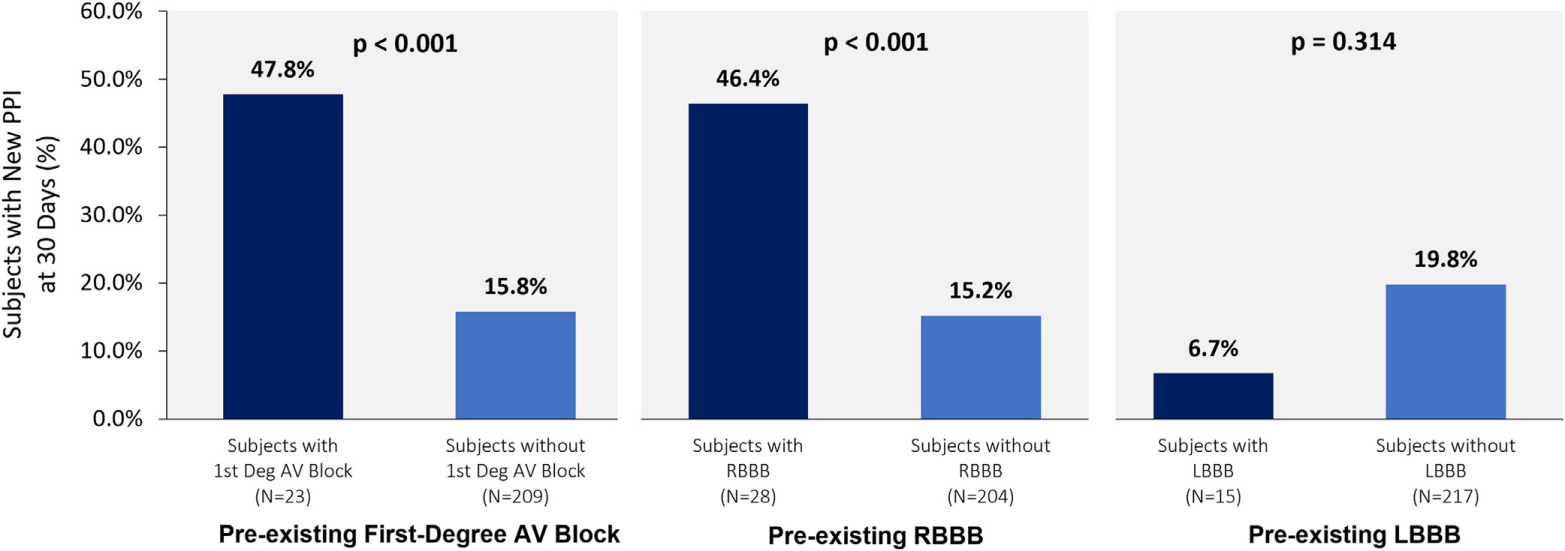
**Prevalent baseline conduction abnormalities.** Patients with pre-existing first-degree AV block and RBBB had significantly higher rates of new PPI post-TAVR compared to patients without. There was no significant difference between patients with or without LBBB and new PPI post-TAVR. Abbreviations: AV, atrioventricular; LBBB, left bundle branch block; PPI, permanent pacemaker implantation; RBBB, right bundle branch block; TAVR, transcatheter aortic valve replacement.

An analysis was performed to assess whether there was a significant difference between patients with vs. without baseline conduction abnormalities known to be associated with new PPI post-TAVR, as presented in Figure 1. Patients with pre-existing first-degree AV block (47.8 vs. 15.8%, *p* < 0.001) and RBBB (46.4 vs. 15.2%, *p* < 0.001) had higher rates of new PPI compared to patients without first-degree AV block or RBBB. There was no significant difference between patients with vs. without pre-existing LBBB (6.7 vs. 19.8%, *p* = 0.314) and new PPI.

### Multivariable Logistic Regression Analysis to Determine Predictors for New PPI

A multivariable model with baseline and procedural covariates was used to examine independent predictors of new PPI post-TAVR. The list of covariates included in the model is presented in Supplemental Table 1. Seven covariates were selected from the univariable analysis for incorporation into the multivariable analysis: pre-existing conduction disturbance, implant depth on the NCC, MS length ≤NCC implant depth, resheathing, QRS interval >120 ​ms, aortic annulus eccentricity ≥0.73, and aortic valve area. Only 1 variable, MS length ≤NCC implant depth (*p* = 0.0023, odds ratio of 3.42), was selected by the model as significant in the multivariable analysis.

**Figure 2:**
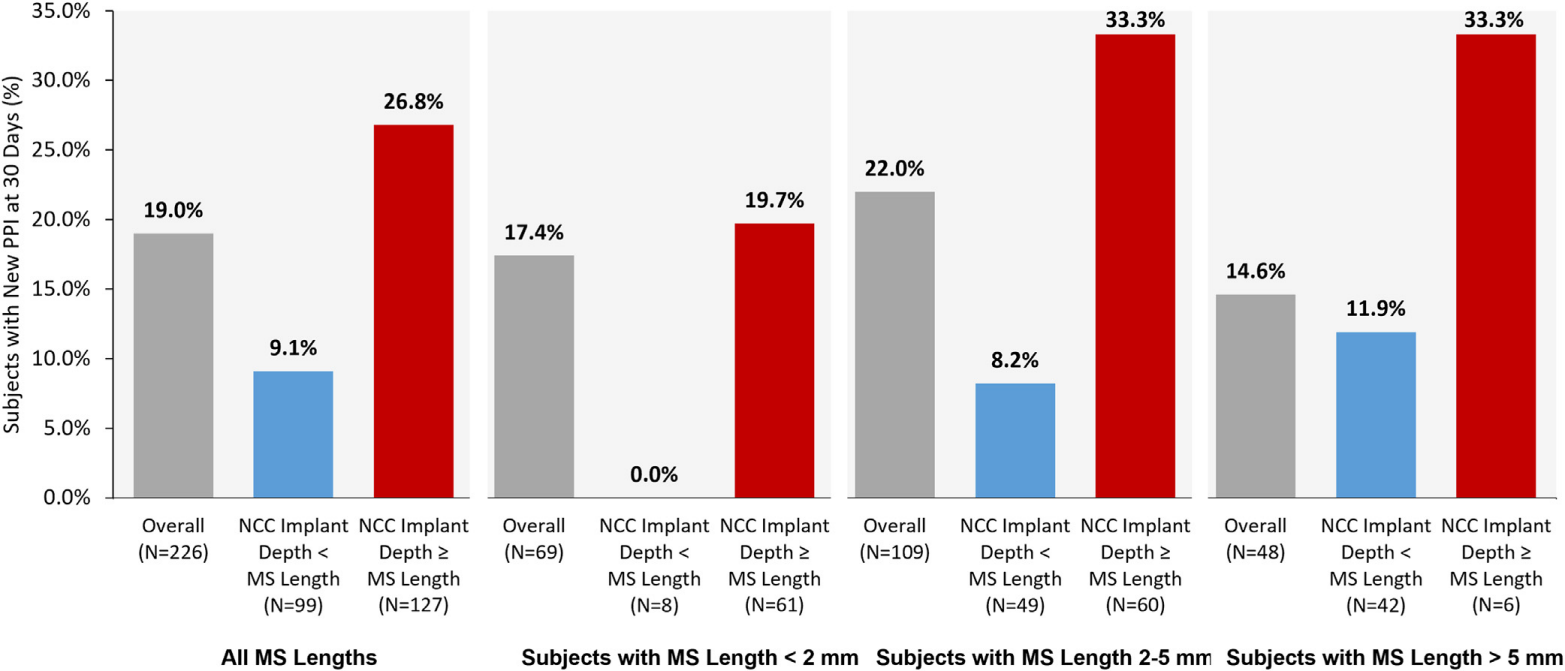
**The impact of new PPI when depth of implant relative to MS length is considered.** The rate of new PPI was numerically higher overall and in all MS length groups when NCC implant depth was greater than or equal to MS length. Grey = Overall new PPI rate in subgroup. Blue = New PPI rate in subgroup when NCC implant depth is less than MS length. Red = New PPI rate in subgroup when NCC implant depth is greater than or equal to MS length. Abbreviations: MS, membranous septum; NCC, noncoronary cusp; PPI, permanent pacemaker implantation.

MS length and its relation to NCC implant depth and new PPI are presented in Figure 2. MS length was not available for 6 patients. The overall rate of new PPI when NCC implant depth was greater than or equal to MS length was 26.8 vs. 9.1% when NCC implant depth was less than MS length (see Panel 1).

Additionally, patients were grouped based on length of MS: short (Panel 2; <2 mm, n = 69), medium (Panel 3; 2-5 mm, n = 109), and long (Panel 4; >5 mm, n = 48). In all subgroups, the rate of new PPI was higher when NCC implant depth was greater than or equal to MS length. Excluding patients with pre-existing first-degree AV block and RBBB from the analysis, the rate of new PPI was <5% in all subgroups when NCC implant depth was less than MS length (Supplemental Figure 1).

### NCC Implant Depth

**Figure 3:**
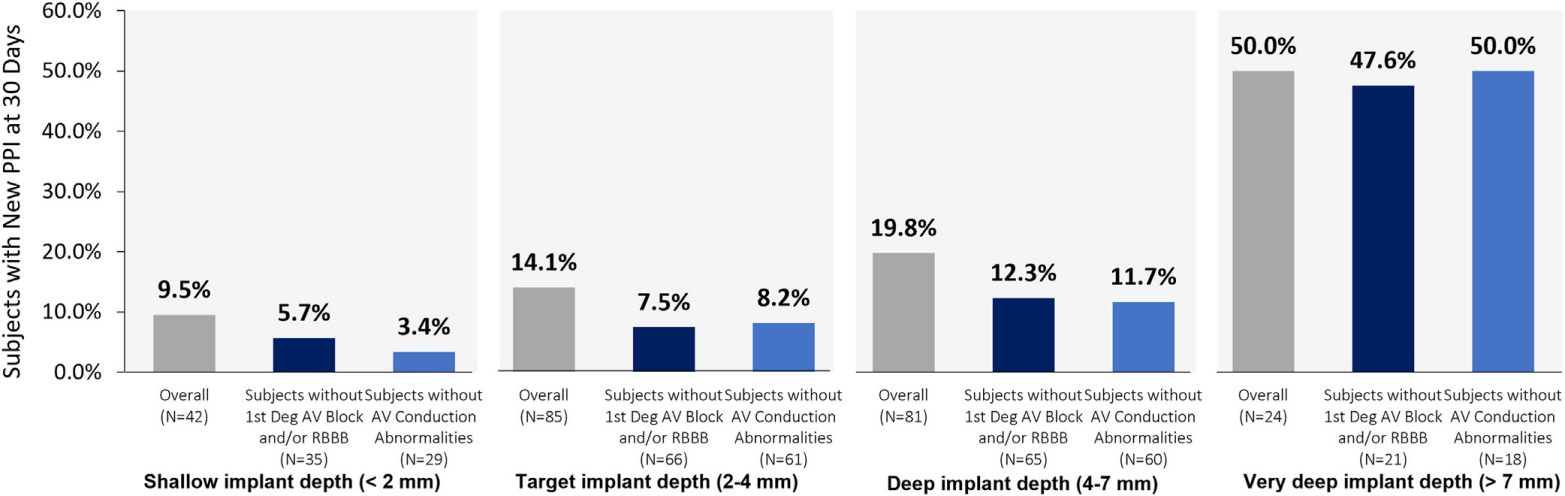
**Valve implant depth alone impacts the need for new PPI.** The rate of new PPI increased with increased NCC implant depth. When excluding patients with pre-existing first-degree AV block or RBBB, the rate of new PPI was <10% in the target and shallow subgroups. When excluding patients with any conduction abnormality, the rate of new PPI remained similar. Gray = Overall new PPI rate in subgroup. Dark blue = New PPI rate in subgroup excluding patients with first-degree AV block or RBBB. Light blue = New PPI rate in subgroup excluding all patients with baseline conduction abnormalities. Abbreviations: AV, atrioventricular; NCC, noncoronary cusp; PPI, permanent pacemaker implantation; RBBB, right bundle branch block.

The relationship of implant depth to new PPI, irrespective of MS length, is presented in Figure 3. All pacemaker-naïve patients had a recorded NCC implant depth. The overall rate of new PPI was 14.1% when the Navitor valve was implanted in the target range (Panel 2), 9.5% when implanted shallow (Panel 1), 19.8% when implanted deep (Panel 3), and 50.0% when implanted very deep (Panel 4). Excluding patients with pre-existing first-degree AV block and/or RBBB (dark blue bars), the rate of new PPI was <10% in both the target (7.5%) and shallow (5.7%) subgroups, but >10% in the deep (12.5%) and very deep (47.6%) subgroups. Excluding all patients with pre-existing conduction abnormalities (light blue bars), the rate of new PPI remained similar in each subgroup vs. when only patients with first-degree AV block and/or RBBB were removed.

## Discussion

**Figure 4:**
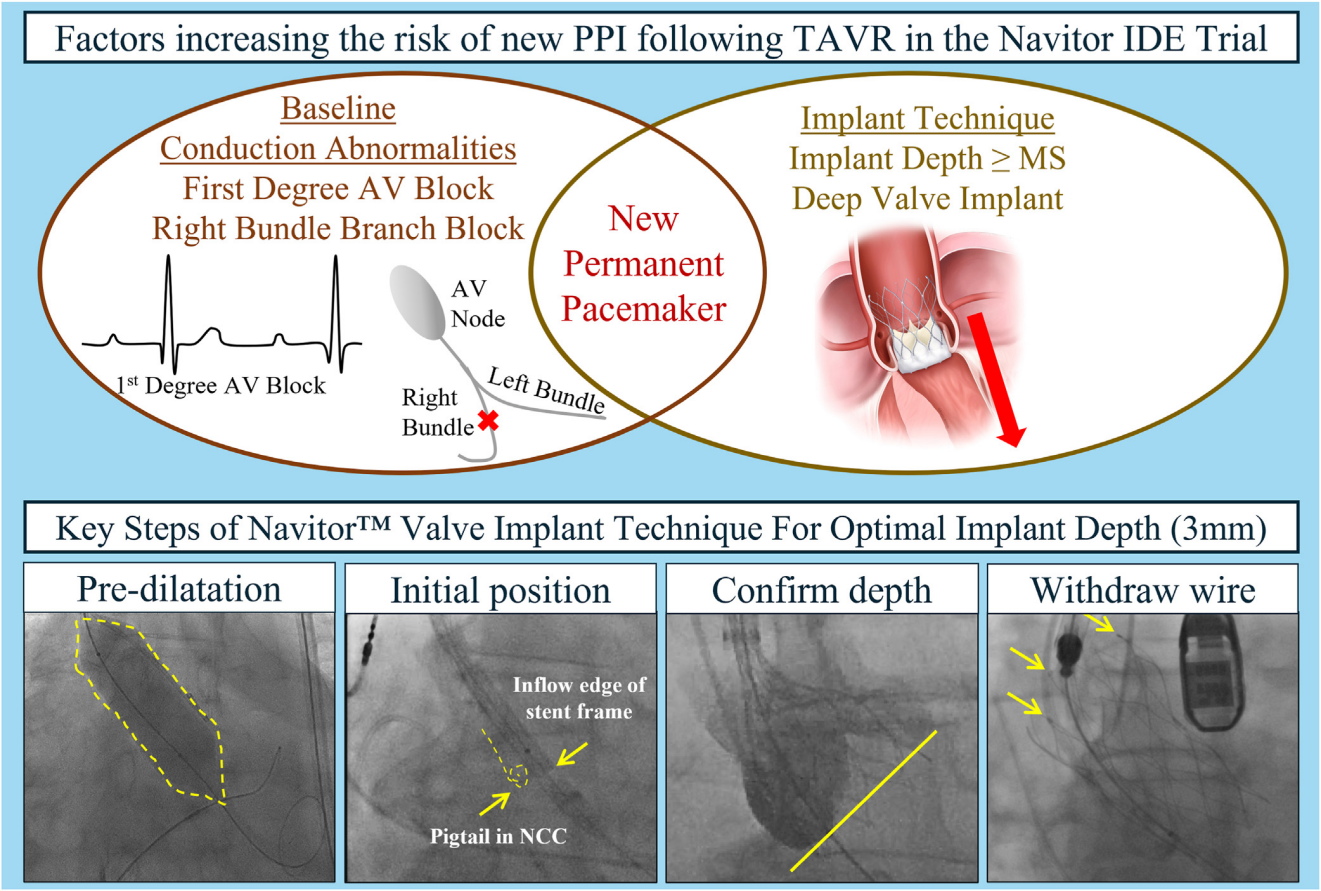
**Factors increasing the risk of new PPI following TAVR in the Navitor IDE Trial.** Baseline conduction disturbances, namely first-degree AV block and RBBB, as well as implant depth, increase the risk of new PPI following TAVR. To prevent deep implantation of the Navitor valve, Abbott has refined the implant technique to attain a target implant depth of 3 mm. Key steps include: 1) predilatation to prepare the annulus for the TAVR procedure; 2) position the inflow edge of the stent frame so it is aligned with the base of the NCC; 3) confirm implant depth in cusp overlap and an alternate view to ensure a target implant depth of 3 mm (recommended per IFU); and 4) withdraw guidewire to a mid-ventricular position and confirm detachment of all three retainer tabs. Abbreviations: AV, atrioventricular; IFU, instructions for use; MS, membranous septum; NCC, noncoronary cusp; PPI, permanent pacemaker implantation; RBBB, right bundle branch block; TAVR, transcatheter aortic valve replacement.

We report a detailed investigation into new PPI post-TAVR for the Navitor valve in the Navitor IDE study. The results from these analyses suggest baseline conduction abnormalities and implant depth were associated with the rate of new PPI (Figure 4).

### 30-Day New PPI Rate in Context

The rate of new PPI at 30 days was 19.0% in the Navitor IDE study.^5^ In other premarket studies of next-generation TAVR devices (i.e., with a paravalvular leak mitigation feature), the rate of new PPI is numerically lower and ranges from 11.8 to 15.0% in a similar high- or extreme-risk population.^9^, ^10^, ^11^ However, in a postmarket setting, the rate of new PPI varies widely. For example, the FORWARD PRO registry reported a 30-day new PPI rate of 20.7%, hypothesized to be attributed to a large portion of patients (30%) with pre-existing conduction disturbances.^12^ The ACURATE neo2 PMCF study and SOURCE 3 registry reported 30-day new PPI rates lower than their respective premarket rates of 6.5 and 12.0%.^13^^,^^14^ Due to the variation of PPI rates across studies, either due to sample size, chance, or other factors, cross-study comparisons should be considered with caution.

### Baseline Conduction Disturbance Is Associated With New PPI

Of the 232 pacemaker-naïve patients, 64 (27.6%) had 1 (or more) of the following baseline conduction abnormalities: first-degree AV block, second-degree AV block (Type I or II), RBBB, LBBB, LAFB. As most (45/64, 70.3%) pacemaker-naïve patients with baseline conduction abnormalities had either first-degree AV block and/or RBBB, it is important to understand the potential association between these abnormalities and risk for new PPI post-TAVR. In our analysis, patients with first-degree AV block (47.8 vs. 15.8%) or RBBB (46.4 vs. 15.2%) were roughly 3x as likely to receive a new PPI post-TAVR compared to patients without first-degree AV block or RBBB at baseline. This is consistent with reports of both self-expanding and balloon-expandable valves, where RBBB was found to be an important predictor of new PPI.^15^^,^^16^

Indeed, a 2014 meta-analysis of 41 studies that reported incidence of new PPI post-TAVR revealed that pre-existing first-degree AV block was a predictor of PPI in 6 studies, while RBBB was a predictor of PPI in 17 studies.^17^ In the same meta-analysis, LBBB was a predictor of PPI in 16 studies, unlike our findings in the Navitor IDE study. A more recent meta-analysis of 78 studies performed in 2021 confirmed earlier findings, revealing baseline conduction abnormalities, including second-degree AV block Type I, LAFB, and RBBB were associated with higher odds of new PPI post-TAVR.^18^ These meta-analysis data suggest an increased likelihood of new PPI post-TAVR with common baseline AV conduction abnormalities, especially RBBB, irrespective of other factors.

### The Importance of the Membranous Septum

Our predictor analysis revealed NCC implant depth relative to MS length as the only independent predictor of new PPI in the Navitor IDE study. The relative length of the MS (short, medium, or long) did not impact the overall trend observed, as this association was noted across all categories of MS length. This is consistent with other analyses of implant depth and MS length in patients who received a self-expandable TAVR valve,^3^^,^^4^ as well as balloon-expandable TAVR.^19^

Consistent with recent studies, our study shows that MS length is an important patient factor associated with new PPI. Understanding patient-specific cardiac anatomy, specifically measuring the MS, may aid operators in offering better prognostications of the likelihood for the need of a PPI following TAVR. While measuring MS length may allow physicians to have a discussion with patients with regards to the need for pacemaker, it may be challenging to implant with extreme precision. Operators should continue to be cautious about depth of deployment and the need for pacemaker vs. an embolized transcatheter heart valve.

### Implant Technique Best Practices Will Help Achieve Target Implant Depth

This study showed that as the depth of valve implantation increases, irrespective of MS length or baseline conduction disturbances, so too does the rate of new PPI. This phenomenon has also been shown for other self-expanding valve platforms.^15^ This has been hypothesized to be due to the compression of the cardiac conduction system caused by the valve stent frame, leading to unresolvable post-TAVR conduction disturbances.^20^ To prevent deep implantation of the Navitor valve, Abbott has refined the implant technique to attain a target implant depth of 3 mm (recommended in the Instructions for Use). Key steps of this implant technique are demonstrated in Figure 4 and described below.

First, predilatation is recommended to prepare the annulus for the Navitor valve and reduces the need for resheathing and postdilatation, thus limiting procedural manipulations of the valve. Following predilatation, initial valve positioning is critical. Positioning the inflow edge of the stent frame so it is aligned with the base of the NCC allows for optimal positioning, which will aid in achieving the recommended target implant depth. Third, prior to releasing the valve, confirming the implant depth in both cusp overlap and an alternative view ensures proper mitigation of paravalvular leak and reduces the risk of depth-related PPI. Imaging in the cusp overlap view provides an accurate depth of implant for the NCC, while a subsequent left anterior oblique view with parallax removed confirms the depth for the left coronary cusp. Lastly, following valve deployment, withdrawing the guidewire to a mid-ventricular position, and confirming all 3 retainer tabs have detached, ensures no unintentional snaring or movement of the valve.

### Limitations

There are limitations to consider in this study. Implant depth used in these analyses were site-reported and not confirmed by an independent arbitrator. In addition, other factors (i.e., calcium burden, additional procedural manipulations, and new intraprocedural conduction disturbances) have been shown to be associated with new PPI post-TAVR. The authors investigated major signals pertaining to new PPI in this study, and further investigation into other factors influencing new PPI are beyond the scope of this report.

## Conclusions

The rate of new PPI at 30 days in the Navitor IDE study is associated with patient pre-existing baseline conduction disturbances and implant depth. With increased operator experience of the Navitor TAVR system and understanding patient-specific anatomic nuances, the rate of new PPI is expected to diminish in real-world practice.

## Impact on Daily Practice

Informed discussions with patients surrounding pre-existing conduction disturbances and anatomical considerations will allow implanters to inform patients about their risk of receiving a pacemaker following an implant with the Navitor valve.

## Ethics Statement

The study was conducted in accordance with the Declaration of Helsinki and was approved by the appropriate Institutional Review Board/Ethics Committee of each investigational site and by the applicable regulatory authorities. All patients provided informed consent prior to participation.

## Funding

The study was sponsored by Abbott.

## Disclosure Statement

I. Sultan is a consultant and receives research support from Abbott, Artivion, Atricure, Boston Scientific, Edwards Lifesciences, Medtronic, and Terumo Aortic. M. J. Reardon reports consultant fees and/or institutional research grants from Abbott, Boston Scientific, Medtronic, and Gore Medical. L. Sondergaard is Chief Medical Officer at Abbott Structural Heart and has previously received consultant fees and/or institutional research grants from Abbott, Boston Scientific, Medtronic, and Sahajanand Medical Technologies. B. Chehab reports speaker, consultancy, and proctor fees for Abbott, Edwards Lifesciences, Medtronic, and CSI. D. Smith reports speaker and proctor fees for Abbott, Edwards Lifesciences, and Biosensors. A. S. Walton is a proctor on medical advisory boards and receives grant support from Abbott, Medtronic, and Edwards Lifesciences. S. G. Worthley has received speaker fees and proctorship from Abbott and HighLife Medical, as well as consultancy fees from Edwards Lifesciences. G. Manoharan is a proctor for Abbott and Medtronic. G. Yong is a proctor for Abbott. H. Jilaihawi reports consultant fees and/or institutional research grants from Abbott, Edwards Lifeciences, Medtronic, and Pi-Cardia. F. Asch reports institutional research grants from Abbott, Edwards, Medtronic, BSC, Corcym, and Biotronik. N. Bates is an employee of Abbott. G. P. Fontana is a consultant, proctor, and speaker for Abbott and Medtronic.
