# Supplementary material for: Predictors and Trends of New Permanent Pacemaker Implantation: A Subanalysis of the International Navitor IDE Study
Source: Struct Heart. 2024 Mar 23;8(4):100293. doi: 10.1016/j.shj.2024.100293 (PMC11294828; doi:10.1016/j.shj.2024.100293)
Supplement: Supplementary figure and Table [file mmc1.docx]

Supplemental Appendix

Title: Predictors and Trends of New Permanent Pacemaker Implantation: A sub-analysis of the international Navitor IDE study

**LIST OF TABLES**

[Table 1. List of Covariates in the Univariable Logistic Regression Analysis 2](#_Toc133308254)

**LIST OF FIGURES**

[Figure 1. The impact of new PPI when depth of implant relative to MS length is considered, excluding subjects with pre-existing first-degree AV block and/or RBBB 3](#_Toc133321458)

A multivariate model was used to assess independent predictors of new PPI post-TAVI. Covariates in the univariable logistic regression analysis included baseline characteristics, medical history, and procedural characteristics. Covariates with significance of p < 0.20 (shaded in grey) were used in the final multivariable logistic regression analysis.

Table 1. List of Covariates in the Univariable Logistic Regression Analysis

| **Covariate** | **OR** | **p Value** |
| --- | --- | --- |
| Pre-TAVI Conduction Disturbance (yes) | 3.476 | 0.0004 |
| Implant depth on non-coronary cusp (mm) | 1.676 | 0.0022 |
| MS Length <= NCC Implant Depth | 3.423 | 0.0023 |
| Resheathing (yes) | 2.032 | 0.0404 |
| QRS interval > 120 ms | 5.333 | 0.0500 |
| Eccentricity >= 0.73 | 0.302 | 0.0500 |
| AVA (cm^2) | 3.589 | 0.1831 |
| Severity of aortic valve Calcification (Moderate, Severe) | 0.549 | 0.2446 |
| Pre-BAV balloon/annulus diameter ratio | 0.039 | 0.2671 |
| Previous atrial fibrillation | 1.504 | 0.2761 |
| Age | 1.027 | 0.4107 |
| PR interval > 200 ms | 2.167 | 0.4177 |
| Mean annulus diameter (mm) | 1.075 | 0.4398 |
| Valve size | 1.074 | 0.4629 |
| Pre-BAV (yes) | 0.607 | 0.4756 |
| Post-BAV (yes) | 1.284 | 0.4838 |
| Sinus bradycardia | 1.177 | 0.8085 |
| Severity of LVOT calcification (Moderate, Severe) | 0.907 | 0.8210 |
| LVOT diameter (mm) | 1.010 | 0.8945 |
| STS Predicted Risk of mortality (%) | 1.010 | 0.9045 |
| Male | 0.976 | 0.9440 |

OR = Odds Ratio

Figure 1. The impact of new PPI when depth of implant relative to MS length is considered, excluding subjects with pre-existing first-degree AV block and/or RBBB


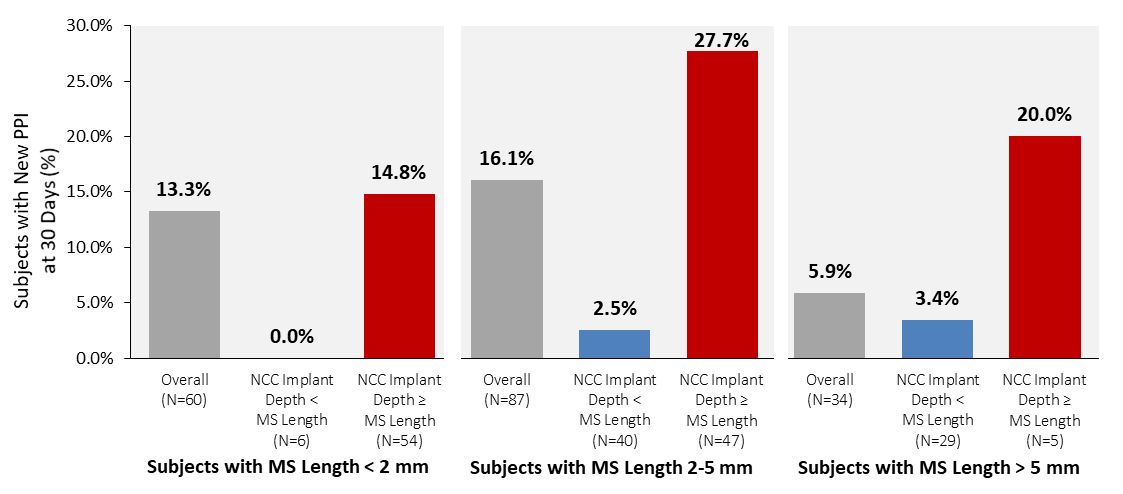


Grey = Overall new PPI rate in subgroup; Blue = New PPI rate in subgroup when NCC implant depth is less than MS length; Red = New PPI rate in subgroup when NCC implant depth is greater than or equal to MS length
